# Processing-Dependent Incorporation of Food By-Product Extract into Zein-Based Active Packaging Films

**DOI:** 10.3390/polym18111347

**Published:** 2026-05-29

**Authors:** Chiara Bufalini, Junyang Li, Emanuela Drago, Alberto Lagazzo, Roberta Campardelli

**Affiliations:** Department of Civil, Chemical and Environmental Engineering, University of Genoa, Via Opera Pia, 15, 16145 Genoa, Italy; chiara.bufalini@edu.unige.it (C.B.); junyangli1314@gmail.com (J.L.); emanuela.drago@unige.it (E.D.); alberto.lagazzo@unige.it (A.L.)

**Keywords:** active food packaging, natural antioxidant extract, non-conventional extraction techniques, bio-based packaging, electrospinning, doctor blade-assisted casting, waste valorization

## Abstract

The recovery of antioxidant compounds from agri-food by-products represents a sustainable strategy for active packaging production. However, the compatibility between natural extracts and film-forming techniques plays a key role in determining film formation and properties. In this work, antioxidant extracts obtained from spent coffee grounds and tomato waste were incorporated into zein-based films produced using two different techniques. The objective was to investigate how extract type and processing technique influence film morphology, wettability, thickness, and mechanical properties. The results demonstrated a strong processing-dependent compatibility between extract composition and production techniques. Spent coffee ground extract was successfully incorporated into compact cast films, while tomato waste extract did not allow the formation of homogeneous cast films and required electrospinning to obtain uniform fibrous structures. The incorporation of spent coffee ground extract significantly increased surface wettability and film stiffness, with Young’s modulus reaching 695 MPa. In contrast, electrospun films containing tomato waste extract exhibited lower tensile strength due to their porous fibrous structure, although uniform fibers were obtained. These findings demonstrate that extract chemistry critically affects the suitability of the processing technique and provide useful guidelines for the design of sustainable zein-based active packaging systems derived from agri-food waste valorization.

## 1. Introduction

Food packaging plays a crucial role in food preservation and safe distribution by protecting products from external contamination and reducing spoilage [1,2,3]. However, the extensive use of petroleum-based plastic materials has generated significant environmental concerns due to waste accumulation and microplastic diffusion [4,5,6]. Consequently, increasing attention has been directed toward the development of sustainable packaging materials derived from renewable resources [7,8,9].

Among the emerging strategies, active packaging represents a promising approach for extending food shelf-life through the incorporation of functional compounds capable of interacting with the packaged food or surrounding environment [10,11,12]. In particular, antioxidant compounds such as polyphenols and carotenoids can limit oxidative degradation phenomena responsible for quality loss in food products [13,14,15,16]. Agri-food by-products, including spent coffee grounds and tomato waste, are attractive sources of these bioactive molecules and offer additional advantages in terms of waste valorization and circular economy approaches [17,18,19].

At the same time, biopolymers have attracted growing interest as sustainable alternatives to conventional plastics. Zein, a corn-derived protein, is particularly attractive due to its biodegradability, film-forming ability, and good oxygen barrier properties [20,21]. Moreover, its amphiphilic nature allows the incorporation of bioactive compounds, although the compatibility between zein and natural extracts strongly depends on extract composition and processing conditions.

The production technique also plays a key role in determining the morphology and final properties of active films. Conventional high-temperature processes may induce degradation of both biopolymers and active compounds [22,23,24]. Therefore, mild processing techniques such as doctor blade-assisted casting and electrospinning are increasingly investigated for the fabrication of bioactive films. Doctor blade-assisted casting enables the production of compact and homogeneous films with controlled thickness [25,26,27,28], whereas electrospinning allows the fabrication of porous fibrous mats characterized by high surface area and tunable morphology [29,30,31,32].

Although several studies have investigated natural extract incorporation into zein-based systems, the relationship between extract composition, processing technique, and final film formation is still not fully understood. In particular, limited information is available regarding the compatibility of phenolic-rich and carotenoid-rich extracts with zein matrices processed through different low-temperature techniques.

Therefore, the aim of this work was to investigate the effect of extract type and processing technique on the formation and properties of zein-based active films. In particular, extracts obtained from spent coffee grounds and tomato waste were incorporated into zein matrices produced by doctor blade-assisted casting and electrospinning. The resulting materials were characterized in terms of morphology, thickness, mechanical properties, wettability, and moisture behavior in order to identify formulation–process relationships relevant for the development of sustainable active packaging systems.

## 2. Materials and Methods

### 2.1. Chemicals

Purified zein, ethanol, glycerol, 2,2′-azino-bis(3-ethylbenzothiazolin-6-sulfonic acid) diammonium salt (ABTS^•+^), Folin–Ciocalteu reagent, and sodium carbonate were purchased from Sigma-Aldrich (Saint Louis, MO, USA). Spent coffee grounds (SCG) were recovered from the vending machine of the Department of Civil, Chemical and Environmental Engineering of the University of Genoa (Italy). Tomato waste (TW) was kindly provided by a producer of tomato sauce in northern Italy.

### 2.2. Extraction of Bioactive Compounds

Spent coffee grounds (SCG) and tomato waste (TW) were selected as agri-food by-product for the extraction of high-added-value compounds. Different methodologies were applied based on previous studies. SCG and TW extracts were selected as representative sources of phenolic and carotenoid antioxidants, respectively, in order to investigate how different extract chemistries affect zein film formation and properties.

The first extract was a polyphenolic extract from SCG. It was extracted using high pressure and temperature extraction (HPTE), following the conditions of Pettinato et al. (2020) [33]. The extraction was performed using a pressure reactor model 4560 (PARR Instrument Company, Moline, IL, USA). The extraction was carried out at 150 °C for 1 h, with solid–liquid ratio of 1:10, under inert atmosphere, and using ethanol 54% *v*/*v*. At the end of the process, the extract was recovered from the exhausted biomass by paper filtration (Whatman 1.2 µm) and stored at −20 °C. The obtained extract was characterized in terms of antiradical power (ARP) and total polyphenolic concentration (TPC).

The extraction of lycopene from TW was performed using ultrasound-assisted extraction. The extraction process was previously optimized by Li et al. (2022) [34]. In detail, the extraction used was composed of an ultrasonic probe (Sonicator Vibra cell 75115, 700 W, Bioblock Scientific Co., Strasbourg, France) equipped with a tip of 13 mm, working at 20 kHz of frequency. The extraction was performed at 65 °C for 20 min using a pulsed mode (30 s on/30 s off) and an amplitude of 65%. The liquid-to-solid ratio was fixed at 72 mL/g. Temperature was continuously monitored by a thermocouple and controlled by a thermostatic bath. After extraction, the liquid phase was separated from the exhausted solid by centrifugation at 6036× *g* for 10 min (MF-20-R, Alliance Bio Expertise, Guipry, France), followed by filtration through a 0.45 µm membrane filter. The resulting extracts were stored at −20 °C. The obtained extract was characterized in terms of total carotenoid concentration (TCC) and total lycopene concentration (TLC).

The antiradical power (ARP) of the SCG extract was evaluated by the ABTS^•+^ assay [35] expressed as micrograms of Trolox equivalent (TE) per volume of extract (mg_TE_/mL). The total polyphenolic concentration (TPC) of SCG extract was determined by Folin–Ciocalteu’s assay [36] and the results were expressed as milligrams of caffeic acid equivalent (CAE) per volume of extract (mg_CAE_/mL). The total carotenoid concentration (TCC) was determined by colorimetric assay and the results were expressed as micrograms of lycopene equivalents (LE) per volume of extract (µg_LE_/mL) [34]. Finally, total lycopene concentration (TLC) for TW extract was determined using a High-Performance Liquid Chromatographer (Agilent 1100 series, Palo Alto, CA, USA), equipped with a C18 (5 µm, 4.6 × 250 mm) reversed phase column (Vydac, Hesperia, CA, USA), and a diode array detector, following the procedure reported by Anguelova et al. (2000) [37]. The results were expressed in terms of micrograms per volume of extract (µg/mL).

### 2.3. Production of Unloaded Zein Film Using Doctor Blade-Assisted Casting and Electrospinning

Polymeric films were produced using doctor blade-assisted casting and electrospinning. For both methods, zein was selected as the polymer to produce films.

For doctor blade-assisted casting, polymeric solutions were prepared by dissolving 2 g of zein in 10 mL of 80% *v*/*v* ethanol aqueous solution, corresponding to a zein concentration of 20% *w*/*v*. Glycerol was added as a plasticizer at a fixed amount of 0.48 mL [38]. The solution was magnetically stirred at 70 °C until complete polymer dissolution was achieved. The resulting solution was deposited onto a glass support and spread using a calibrated doctor blade applicator. The dispensed volume ranged from 5 to 10 mL in order to obtain nominal wet film thicknesses of 0.25 mm and 0.5 mm, respectively. After deposition, the coated substrates were placed horizontally in a ventilated oven at 60 °C to promote controlled solvent evaporation. Drying times were set to 30 min for films cast with a nominal wet thickness of 0.25 mm, and to 75 min for those with a nominal wet thickness of 0.5 mm. Regarding electrospinning, different zein concentrations in the polymeric solution were investigated and glycerol was not added. The solvent consisted of an 80% *v*/*v* ethanol aqueous solution, while zein concentrations of 30 and 40% (*w*/*v*) were tested.

Polymer dissolution was carried out following the same procedure described for cast films. Electrospun films were fabricated at room temperature using an electrospinning setup consisting of a high-voltage power supply, a syringe pump, and a grounded planar collector. Specifically, 6 mL of polymer solution was delivered through a 21-gauge metallic needle acting as the anode at a constant flow rate of 1.2 mL/h. The applied voltage between the needle and the grounded collector was fixed at 17 kV, while the tip-to-collector distance was set at 16.5 cm to promote fiber formation and deposition. The electrospinning process was conducted under previously optimized conditions through preliminary experiments [38]. After deposition, the obtained fibrous mats were stored in a desiccator for 2 h to ensure complete solvent removal. The obtained films were then removed from the support for further characterization.

### 2.4. Production of Loaded Zein Film Using Doctor Blade-Assisted Casting and Electrospinning

Different loaded polymeric films were produced using the obtained natural extracts (SCG and TW extracts). The investigated production techniques were the same as for the empty films (Section 2.3). The extracts were loaded individually in the films to evaluate the effects of different extracts on the film’s characteristics. Extract loading was another analyzed parameter. The selected extract concentrations (10, 20, and 30% *w*/*w* with respect to zein dry weight) were chosen based on preliminary formulation studies and previous literature on zein-based active films loaded with natural extracts [38]. Lower concentrations produced limited observable effects on film properties, while higher concentrations led to formulation instability and poor film processability. The extracts were incorporated directly in their liquid hydroalcoholic form without drying or re-solubilization steps. The final ethanol/water ratio of the film-forming solutions remained approximately consistent with the original hydroalcoholic solvent system used for zein dissolution. The obtained films were removed from the support for further characterizations.

### 2.5. Characterization of Films

#### 2.5.1. Morphology

The morphology of all the produced films was characterized by a field emission scanning electron microscope (FE-SEM, LEO 1525, Carl Zeiss SMT AG, Oberkochen, Germany). The thickness of the films was calculated using a digital micrometer (3791G 0-150, Messzeuge, Spangenberg, Germany). The measurement was performed five times in different zones. In the case of electrospun films, the average fiber diameter and size distributions were evaluated using ImageJ software (Version 1.54p, NIH, Bethesda, MD, USA), randomly analyzing about 300 fibers per sample.

#### 2.5.2. Mechanical Tests

The mechanical properties of the samples were assessed through tensile tests, determining tensile strength (TS), elongation at break (EB), and Young’s modulus (YM). Measurements were carried out using the Zwick Roell Z0.5 equipment in combination with Zwick Roell’s testXpert III software Version 1.4, in accordance with the relevant standards for polymeric materials (UNI EN ISO 527) [39]. Rectangular specimens with dimensions of 10 × 50 mm^2^ were fixed between the machine grips and tested using a 500 N load cell. An initial grip separation of 21 mm and a tensile speed of 10 mm/min. For each formulation, a minimum of five specimens were analyzed to ensure statistical reliability.

#### 2.5.3. Wettability

The surface wettability of the samples was evaluated by contact angle measurements using the sessile drop technique. The tests were carried out at room temperature on zein-based films fixed on suitable support and positioned in front of a digital microscope. A distilled water droplet with a volume of approximately 5 µL was carefully deposited onto the film surface using a micropipette. Images of the droplet profile were acquired immediately after deposition and after 60 s to ensure equilibrium conditions. Contact angle values were determined through image analysis using ImageJ software (NIH, Bethesda, MD, USA) equipped with the “Contact Angle” plug-in. For each sample, at least five measurements were performed, and the results were reported as mean contact angle values ± standard deviation.

#### 2.5.4. Moisture Content

The moisture-related properties of the films were evaluated using a gravimetric method to determine their moisture content and hygroscopic behavior. Approximately 5 mg of each film sample was initially weighed and subsequently dried in a ventilated oven at 60 °C to remove the water contained within the polymer matrix. After the drying process, the samples were reweighed to determine the weight variation associated with water loss. The drying treatment was carried out for 60 min, and measurements were repeated at different time intervals, namely after 0, 7, and 14 days from film preparation, to monitor the evolution of moisture content over time.

### 2.6. Statistics

Statistical analysis was conducted using one-way ANOVA followed by Tukey’s HSD test (Statistica v. 8.0, StatSoft, Tulsa, OK, USA). Experiments were performed in triplicate (*n* = 3), and differences were considered significant at *p* < 0.05.

## 3. Results and Discussion

### 3.1. Extract Production and Characterization

The high-added-value compounds were extracted from food waste using non-conventional extraction techniques. The selected biomasses were spent coffee grounds (SCG) and tomato waste (TW), which allowed us to extract different antioxidant compounds, such as polyphenols and carotenoids. The extraction processes were previously optimized and the obtained extracts were in agreement with the previous ones [33,34]. In detail, the extract derived from SCG was characterized by 56.6 ± 6.2 µg_TE_/L antiradical power (ARP) and 3.6 ± 0.1 mg_CAE_/mL total polyphenolic concentration (TPC). The TW extract was characterized by 27.6 ± 0.28 total carotenoid concentration (TCC) and 21.3 ± 0.7 µg/mL total lycopene concentration (TLC). The use of non-conventional extraction techniques allowed us to use green solvent, like hydroalcoholic solution, that can be immediately loaded in the films, avoiding any pretreatment. The different composition of the two extracts is expected to play a key role in their interaction with the zein matrix. In particular, the SCG extract is rich in phenolic compounds bearing polar functional groups, which may enhance hydrogen bonding interactions with zein and increase film hydrophilicity. In contrast, the TW extract is mainly characterized by carotenoids, which are more hydrophobic and may lead to different compatibility and distribution within the polymer system.

### 3.2. Unloaded Zein Film Produced with Doctor Blade-Assisted Casting

**Figure 1:**
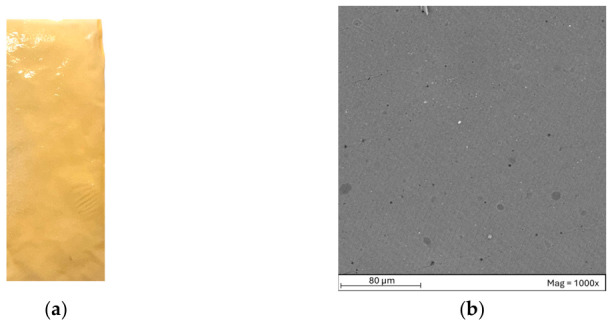
Macroscopic aspect of Z50 (**a**) and FE-SEM image of Z50 (**b**).

The first phase focused on optimizing unloaded zein-based films to find the optimal thickness for good mechanical properties suitable for use in the packaging sector. Specifically, the objective was to achieve a tensile strength greater than 3.5 MPa [40,41] without compromising flexibility. The aim was to balance these different aspects in order to have a robust film that is also capable of withstanding deformation. This is essential for practical packaging applications. The films were produced through doctor blade-assisted casting, which allows greater control of thickness and produces a more uniform and homogeneous film than the classic solvent casting technique, used in previous work [38]. The films were obtained by spreading a known volume of polymer solution and testing two different process thicknesses: 0.25 mm and 0.50 mm. This allowed the production of two different films: Z25 and Z50. The macroscopic aspect and the FE-SEM image of Z50 are reported in Figure 1 as an example. FE-SEM observations revealed a compact and homogeneous morphology without visible pores, aggregates, or phase-separated domains. The films exhibited a continuous structure typical of solvent-cast zein matrices.

Moreover, the films obtained (Z25 and Z50) were characterized in terms of thickness and mechanical properties (Table 1). The thickness of dried films was reduced by 90% compared to the wet ones; this is in line with the solvent evaporation and film densification. Moreover, the thickness was highly uniform in all the film surfaces. The tensile strength (TS) was above the tensile strength limit of food packaging for both films. Conversely, the elasticity was strictly related to the thickness. In fact, thicker films resulted in a more flexible material, with a lower Young’s modulus (YM) and significantly higher elongation at break (EB) than in the case of lower thickness. This combination of strength, elasticity and uniformity has made thicker films preferable for applications requiring durability and mechanical reliability. Furthermore, in comparison to results previously obtained with conventional solvent casting, the doctor blade-assisted casting significantly improved the elasticity of the material produced, which, at the same thickness of approximately 50 µm, increases from 0.6 ± 0.1% to 68.9 ± 34.4% for EB and from 1806.0 ± 368.2 MPa to 389.2 ± 102.9 MPa for YM [38]. The ability to control the deposition of the solution has allowed for a film that is not only more uniform and homogeneous, but also more elastic, while maintaining its strength virtually unchanged. The improved elongation at break observed for thicker films may be related to a more uniform solvent evaporation and a more homogeneous distribution of glycerol within the matrix, which likely promoted polymer chain mobility. This result suggests that doctor blade-assisted casting allowed better control over film formation, reducing structural defects and improving the balance between strength and flexibility.

Based on the results obtained, a thickness of 0.5 mm was chosen for extracts loading, as it is more elastic but still offers good resistance.

### 3.3. Loaded Zein Film Produced with Doctor Blade-Assisted Casting

**Figure 2:**
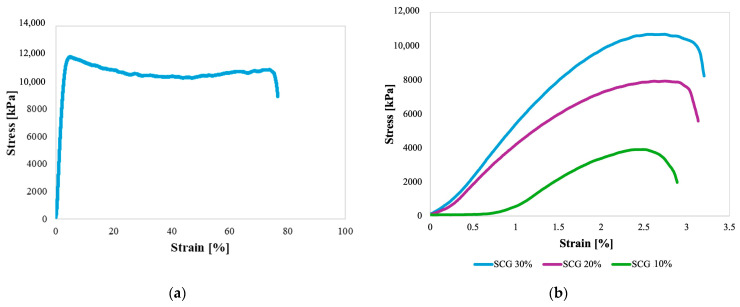
Stress–strain curves of the specimens for the unloaded film (**a**) and for loaded film with 10%, 20% and 30% *w*/*w* of SCG extract (**b**).

Based on the results obtained from the feasibility study on unloaded films, 0.5 mm was selected as the ideal thickness for the production of loaded films. The first extract tested was obtained from spent coffee grounds (SCG), with the aim of obtaining an activated film with antioxidant properties to prolong shelf life. Several loadings were performed: 10, 20 and 30% (*w*/*w*). The objective was to evaluate how the variation in extract concentration affects the mechanical properties of the film, with particular attention to tensile strength and elasticity. The results of the mechanical tests are shown in Table 2 and Figure 2. The obtained results show that, compared to the unloaded zein film, the incorporation of SCG caused a marked change in the mechanical behavior. In particular, all samples containing SCG showed a drastic reduction in elongation at break compared to the control, indicating a significant loss of deformability and greater fragility of the film. This effect was accompanied by an increase in Young’s modulus, which increases progressively with the concentration of the extract, suggesting an increasing rigidity of the polymer matrix. Tensile strength showed a concentration-dependent trend: while at low extract percentages there was a decrease compared to the sample without extract, at higher concentrations the values were comparable or higher than those of the control. Overall, SCG induced a stiffer and less ductile behavior, likely due to reduced mobility of zein chains. Compared to solvent casting, tensile strength was slightly lower in some cases, while elongation at break and Young’s modulus suggested a more elastic response. This behavior may be related to the presence of phenolic compounds, which could interact with zein chains through secondary intermolecular interactions and reduce polymer chain mobility, leading to a stiffer and less deformable structure. At higher extract loadings, the dispersed extract phase may additionally contribute to a reinforcing effect, partially compensating for the initial reduction in tensile strength observed at lower concentration.

In addition to mechanical properties, the contact angle was also evaluated, and the obtained results are reported in Table 3. The unloaded film was characterized by the typical hydrophilicity in presence of glycerol as plasticizer [42,43]. Compared to the unloaded film, the addition of SCG extract resulted in a reduction in the contact angle already at the initial time, indicating a significant increase in the surface hydrophilicity of the material. The contact angle value changed from approximately 50° in the control sample to values below 30° in films containing SCG extract, with a slight further decrease as the concentration increases. The reduction in the contact angle was also observed after 60 s. This marked reduction in contact angle confirms that SCG extract modified the surface chemistry of the films, likely due to the introduction of polar group from phenolic components. This effect adds to the intrinsic hydrophilizing action of glycerol, resulting in faster spreading of water droplets on the film surface. No complete dissolution of the films was observed during contact angle measurements. However, partial local swelling and rapid spreading of the water droplet occurred, particularly for films containing the highest SCG concentration. This behavior may be advantageous for applications requiring rapid interaction with aqueous environments or controlled release of active compounds. However, excessive surface hydrophilicity could represent a limitation for applications requiring high water resistance [44].

**Figure 3:**
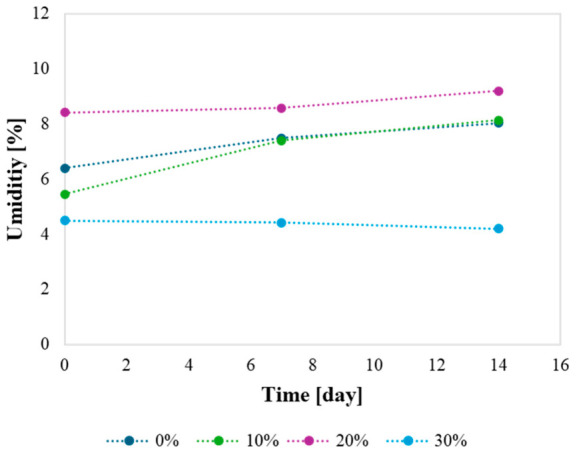
Trend of moisture content of the loaded film with 10%, 20% and 30% *w*/*w* of SCG extract.

Finally, the moisture content of films was evaluated over time, and the obtained trends are reported in Figure 3. The moisture content of the investigated films exhibited limited variations over the storage period, with values remaining within a narrow range for all formulations. Overall, the comparable moisture values recorded at different time points suggest that the films maintain stable hygroscopic properties during storage, confirming the absence of progressive drying or moisture accumulation phenomena. Despite the increased surface hydrophilicity induced by SCG extract, the overall moisture content remained relatively stable over time. This suggests that the extract mainly affected surface wettability rather than causing substantial changes in bulk water uptake during storage. These results indicate that surface wettability and bulk hygroscopicity should not be considered equivalent properties, since surface interactions with water may increase without necessarily inducing significant bulk water absorption.

After loading the SCG extract, tomato waste (TW) extract was loaded. In this case, only 20% and 30% *w*/*w* loadings were investigated. In contrast to the SCG extract, the films loaded with TW extract using the doctor blade-assisted casting exhibited inadequate formation. The films were characterized by insufficient uniformity, both in terms of thickness and in terms of extract distribution. Based on the macroscopic aspect of the sample, an incompatibility was observed between the extract and zein using the doctor blade-assisted casting, which made it impossible to characterize the resulting films. The poor film formation observed with TW extract indicates a limited compatibility between this extract and the casting formulation. This behavior may be related to phase separation phenomena, uneven solvent evaporation, or unfavorable interactions between carotenoid-rich compounds and the zein/glycerol system. This result highlights that extract incorporation cannot be considered independently of the processing route, and that formulation–process compatibility is a crucial design parameter for active bio-based films.

### 3.4. Loaded Zein Film Produced with Electrospinning

Considering the incompatibility between TW extract and doctor blade-assisted casting, the loading of extracts from TW in films produced by electrospinning was also investigated, as the loading of SCG extract in electrospun films was characterized by good results [38].

**Figure 4:**
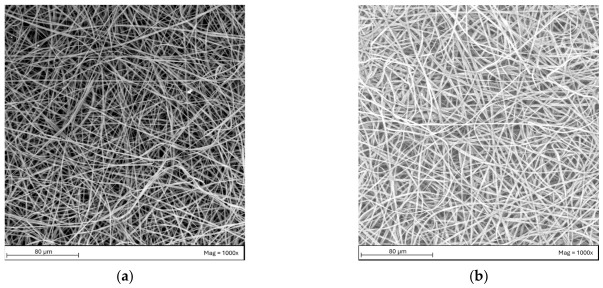

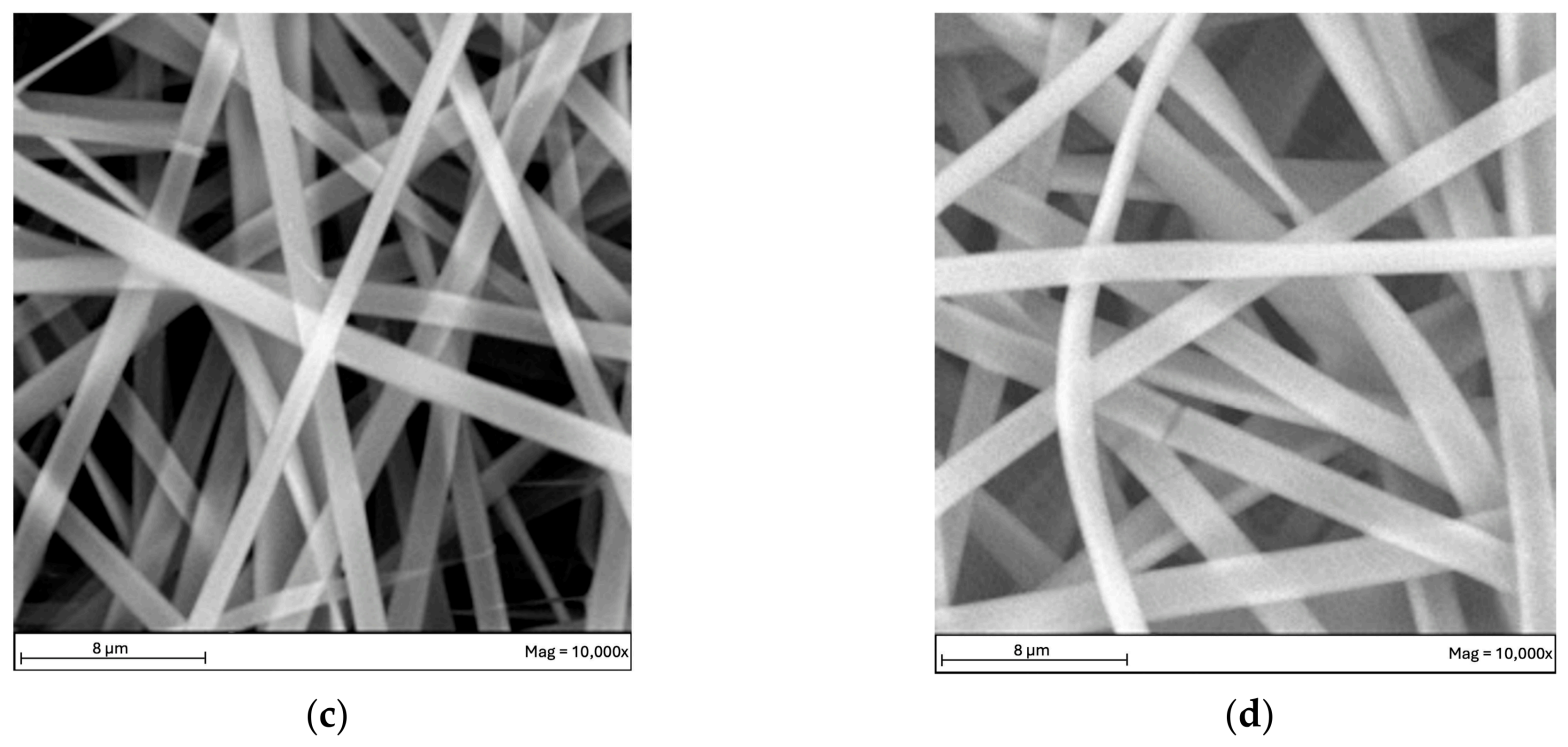
FE-SEM image of unloaded electrospun films at different magnification with 30% *w*/*w* (**a**,**c**) and 40% *w*/*w* (**b**,**d**) of zein concentration.

Initially, unloaded films were produced by varying the concentration of zein in the polymer solution. The films obtained were observed under FE-SEM to examine their morphology (Figure 4). From the images, it can be seen that the lower concentration solution (30%) formed finer and thinner fibers, with an average diameter of 1.4 ± 0.2 µm. In contrast, the fibers produced with a concentration of 40% *w*/*w* had an increase in average fiber diameter, equal to 1.7 ± 0.5 µm. Both films were characterized by random and isotropic orientation of the fibers, which exhibited a ribbon-like structure.

**Figure 5:**
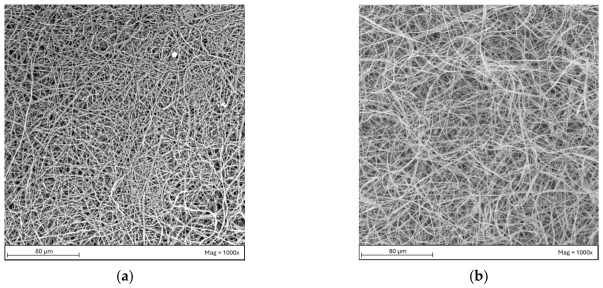

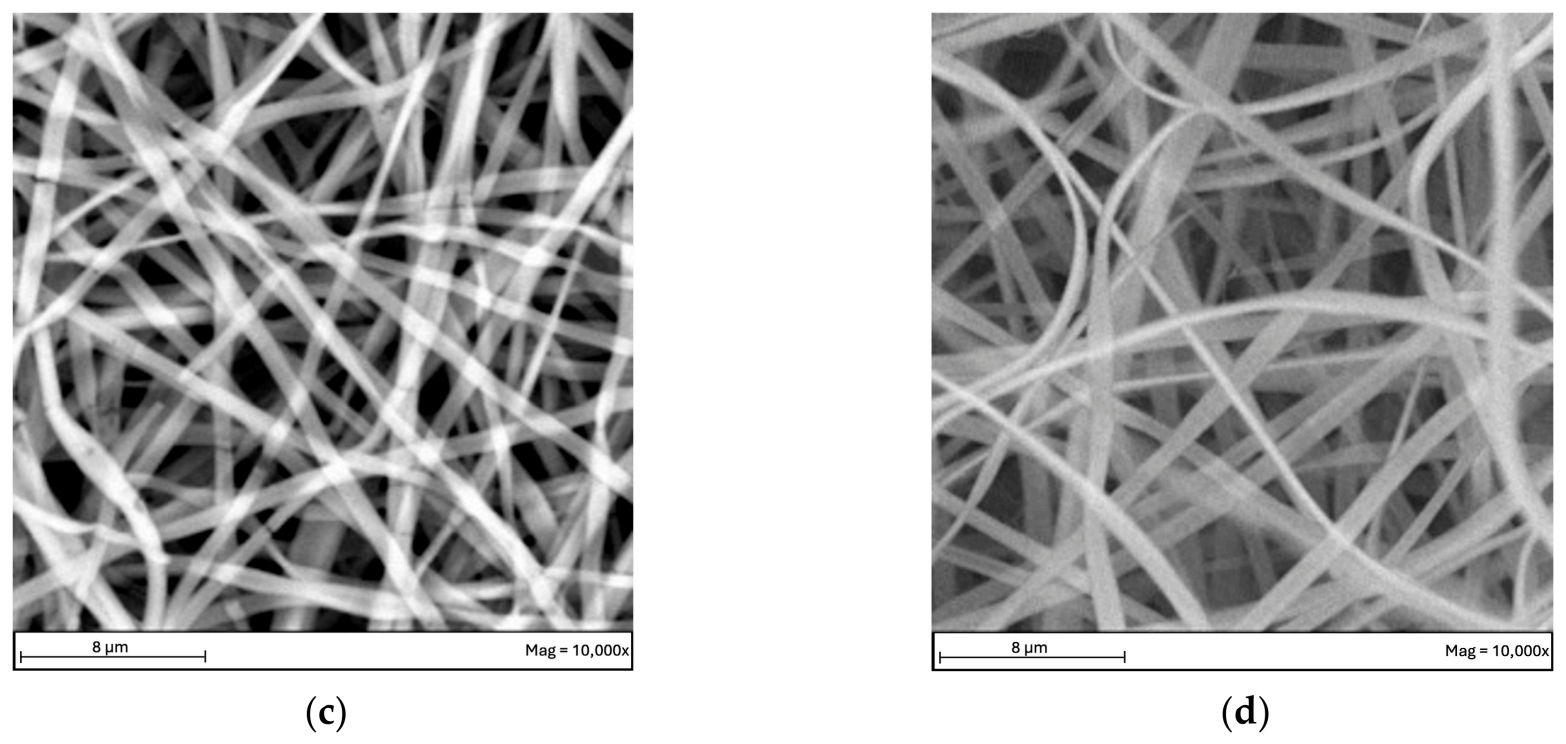
FE-SEM image of natural extract-loaded electrospun films at different magnification with 30% *w*/*w* (**a**,**c**) and 40% *w*/*w* (**b**,**d**) of zein concentration.

Given the good results obtained in the production of unloaded films, both concentrations were investigated for the addition of TW extract (30% *w*/*w*). The loaded films were produced correctly, with the typical appearance of non-woven fabric. The obtained films were characterized using FE-SEM (Figure 5), where it can be seen that the morphology of the ribbon fibers did not undergo significant changes with extract addition. With regard to diameters, it was observed that they were thinner than those of the non-enriched solutions. Specifically, the average diameters measured were 0.9 ± 0.1 µm for the solution containing 30% *w*/*w* zein and 0.9 ± 0.2 µm for the solution containing 40% *w*/*w* zein. The same effect was observed also for the SCG extract [38]. This reduction in fiber diameter after extract addition suggests that the presence of TW extract altered solution properties relevant to electrospinning, such as viscosity, electrical conductivity, or surface tension, thereby affecting jet stretching and fiber formation. Moreover, the addition of TW extract significantly affects the thickness (Table 4). In both cases (30% and 40%), samples containing TW extract showed a reduction in thickness compared to the corresponding unloaded films, suggesting that the presence of the extract may modify the viscosity of the solution or the deposition process during electrospinning, leading to more compact structures.

All the films produced were also characterized in terms of mechanical properties, as reported in Table 4. The elasticity of the loaded film was slightly improved compared to the unloaded film in the case of 30% *w*/*w* zein. Regarding the tensile strength, the value did not reach the tensile strength limit required for food packaging. The lower tensile strength of electrospun films compared to cast films is consistent with their non-woven fibrous architecture, which is typically more porous and mechanically weaker than compact continuous films. Nevertheless, electrospinning enabled the successful incorporation of TW extract, which could not be processed by casting, confirming its suitability for systems with lower casting compatibility.

## 4. Conclusions

In this work, antioxidant extracts recovered from agri-food by-products were successfully used as functional additives in zein-based films for active packaging applications. The results demonstrated that the successful incorporation of natural extracts into zein films strongly depends on the compatibility between extract composition and processing technique, highlighting that extract chemistry critically affects process selection. In particular, spent coffee ground extract was effectively incorporated into compact films produced by doctor blade-assisted casting, whereas tomato waste extract did not allow the formation of homogeneous cast films and required electrospinning to obtain uniform fibrous structures. This result emphasizes the importance of formulation–process compatibility in the design of bio-based active packaging systems and provides practical guidelines for future applications involving natural extracts recovered from agri-food waste. The incorporation of SCG extract significantly affected the physico-chemical properties of the cast films. In particular, SCG-loaded films showed increased surface wettability, as indicated by the reduction in water contact angle, and a progressively stiffer mechanical behavior with increasing extract loading. This was evidenced by the increase in Young’s modulus and tensile strength, together with the low elongation at break values, suggesting reduced chain mobility and a more rigid zein network. On the other hand, electrospun films loaded with tomato waste extract showed lower tensile strength than compact cast films, mainly due to their porous fibrous architecture, although the extract was successfully incorporated without markedly altering fiber morphology.

Overall, this study highlights that extract incorporation in bio-based polymer films cannot be considered independently of the processing technique, as formulation–process compatibility represents a key parameter in the design of active packaging materials. The combination of different fabrication techniques may enable the development of multilayer or hybrid structures capable of incorporating different bioactive compounds within the same packaging system. Further spectroscopic and thermal analyses will be necessary to fully elucidate the molecular interactions between zein and the incorporated extracts. These findings provide useful guidelines for the development of sustainable active packaging materials based on the valorization of agri-food waste.

## Figures and Tables

**Table 1:** Thickness and mechanical properties of unloaded zein film produced with doctor blade-assisted casting (Z25 and Z50). T: thickness, TS: tensile strength, EB: elongation at break, YM: Young’s modulus. Different letters refer to statistically significant differences among results (*p* < 0.05, ANOVA with Tukey’s HSD post hoc multiple comparison test).

Sample	T [µm]	TS [MPa]	EB [%]	YM [MPa]
Z25	37.0 ± 4.5 ^a^	13.1 ± 4.1 ^a^	15.5 ± 3.7 ^a^	682.7 ± 297.4 ^a^
Z50	52.1 ± 3.7 ^b^	8.7 ± 1.2 ^a^	68.9 ± 9.5 ^b^	389.2 ± 102.9 ^a^

**Table 2:** Thickness and mechanical properties of loaded zein film with SCG (10, 20, 30% *w*/*w*) produced with doctor blade-assisted casting. T: thickness, TS: tensile strength, EB: elongation at break, YM: Young’s modulus. Different letters refer to statistically significant differences among results (*p* < 0.05, ANOVA with Tukey’s HSD post hoc multiple comparison test).

Sample	T [µm]	TS [MPa]	EB [%]	YM [MPa]
10%	70.1 ± 4.9 ^a^	4.4 ± 0.7 ^a^	2.6 ± 0.5 ^a^	397.0 ± 78.3 ^a^
20%	62.0 ± 10.2 ^ab^	8.8 ± 0.8 ^b^	4.0 ± 0.7 ^a^	615.3 ± 68.5 ^b^
30%	56.3 ± 6.9 ^b^	10.7 ± 1.7 ^b^	3.2 ± 2.4 ^a^	694.6 ± 85.0 ^b^

**Table 3:** Contact angle (CA) obtained from empty (0%) and loaded film with different percentages of SCG (10, 20, 30% *w*/*w*) at instant zero (t = 0 s) and after 60 s (t = 60 s). ND: not detectable.

Sample	CA at t = 0 s	CA at t = 60 s
0%	50.4 ± 6.21°	45.0 ± 7.49°
10%	27.9 ± 3.59°	25.7 ± 1.85°
20%	23.1 ± 1.94°	21.2 ± 3.36°
30%	23.5 ± 1.88°	ND

**Table 4:** Thickness and mechanical properties of empty and loaded zein film with TW with different zein concentration (30 and 40% *w*/*w*) produced with electrospinning. T: thickness, TS: tensile strength, EB: elongation at break, YM: Young’s modulus.

Sample	T [µm]	TS [MPa]	EB [%]	YM [MPa]
30%	88 ± 13.4	1.2 ± 0.1	1.0 ± 0.2	299.4 ± 39.7
30% TW	49 ± 9.9	0.4 ± 0.1	2.0 ± 0.9	198.5 ± 55.6
40%	73 ± 8.9	1.0 ± 0.2	0.5 ± 0.1	301.5 ± 71.7
40% TW	46 ± 7.0	1.7 ± 0.4	0.6 ± 0.1	451.2 ± 54.1

## Data Availability

The original contributions presented in this study are included in the article. Further inquiries can be directed to the corresponding author.

## Abbreviations

The following abbreviations are used in this manuscript:

ABTS^•+^	2,2′-azino-bis(3-ethylbenzothiazolin-6-sulfonic acid) diammonium salt
SCG	Spent coffee grounds
TW	Tomato waste
HPTE	High pressure and temperature extraction
ARP	Antiradical power
TPC	Total polyphenolic concentration
TCC	Total carotenoids concentration
TLC	Total lycopene concentration
TE	Trolox equivalent
CAE	Caffeic acid equivalent
LE	Lycopene equivalents
FE-SEM	Field emission scanning electron microscope
Z25	Films with process thicknesses of 0.25 mm
Z50	Films with process thicknesses of 0.50 mm
TS	tensile strength
YM	Young’s modulus
EB	Elongation at break
CA	Contact angle
ND	Not detectable

## References

[1] Hassoun A., Boukid F., Ozogul F., Aït-Kaddour A., Soriano J.M., Lorenzo J.M., Perestrelo R., Galanakis C.M., Bono G., Bouyahya A. (2023). Creating New Opportunities for Sustainable Food Packaging through Dimensions of Industry 4.0: New Insights into the Food Waste Perspective. Trends Food Sci. Technol..

[2] Maurizzi E., Bigi F., Quartieri A., De Leo R., Volpelli L.A., Pulvirenti A. (2022). The Green Era of Food Packaging: General Considerations and New Trends. Polymers.

[3] Brennan L., Langley S., Verghese K., Lockrey S., Ryder M., Francis C., Phan-Le N.T., Hill A. (2021). The Role of Packaging in Fighting Food Waste: A Systematised Review of Consumer Perceptions of Packaging. J. Clean. Prod..

[4] Versino F., Ortega F., Monroy Y., Rivero S., López O.V., García M.A. (2023). Sustainable and Bio-Based Food Packaging: A Review on Past and Current Design Innovations. Foods.

[5] Siracusa V., Rosa M.D. (2018). Sustainable Packaging. Sustainable Food Systems from Agriculture to Industry.

[6] Scherhaufer S., Moates G., Hartikainen H., Waldron K., Obersteiner G. (2018). Environmental Impacts of Food Waste in Europe. Waste Manag..

[7] Davidescu M.A., Pânzaru C., Mădescu B.M., Poroșnicu I., Simeanu C., Usturoi A., Matei M., Doliș M.G. (2025). Advances and Challenges in Smart Packaging Technologies for the Food Industry: Trends, Applications, and Sustainability Considerations. Foods.

[8] Kadirvel V., Palanisamy Y., Ganesan N.D. (2025). Active Packaging System—An Overview of Recent Advances for Enhanced Food Quality and Safety. Packag. Technol. Sci..

[9] Silva E.G.S., Cardoso S., Bettencourt A.F., Ribeiro I.A.C. (2022). Latest Trends in Sustainable Polymeric Food Packaging Films. Foods.

[10] Lou W., Huang Z., Shao Q., Shan Y., Shi D., Chen Z., Zhang J., Yu W., Wang J., Yang H. (2025). Recent Advances in Active Packaging: Insights into Novel Functional Elements, Response Strategies and Applications for Food Preservation. Food Packag. Shelf Life.

[11] Vilela C., Kurek M., Hayouka Z., Röcker B., Yildirim S., Antunes M.D.C., Nilsen-Nygaard J., Pettersen M.K., Freire C.S.R. (2018). A Concise Guide to Active Agents for Active Food Packaging. Trends Food Sci. Technol..

[12] Wyrwa J., Barska A. (2017). Innovations in the Food Packaging Market: Active Packaging. Eur. Food Res. Technol..

[13] Cazón P., Mateus A.R., Silva A.S. (2025). Advances in Active Packaging Using Natural Biopolymers and Fruit By-Products for Enhanced Food Preservation. Food Res. Int..

[14] Deshmukh R.K., Hakim L., Gaikwad K.K. (2023). Active Packaging Materials. Curr. Food Sci. Technol. Rep..

[15] Singh A.K., Kim J.Y., Lee Y.S. (2022). Phenolic Compounds in Active Packaging and Edible Films/Coatings: Natural Bioactive Molecules and Novel Packaging Ingredients. Molecules.

[16] Eze C.N., Aduba C.C., Ezema B.O., Ayoka T.O., Nnadi C.O., Onyeaka H. (2025). The Role of Antioxidants in Food Safety and Preservation: Mechanisms, Applications, and Challenges. Cogent Food Agric..

[17] Dhar P., Raj B.J.R., Dasarathy A.K., Das P., Singhe S., Palanivelu E., Sinha A., Puppala R., Abate L. (2026). Bioactive Compounds From Agri-Food By-Products: Advancements in Environmental Sustainability and Bioeconomic Progress. Eng. Rep..

[18] Shawky E., Gibbons S., Selim D.A. (2025). Bio-Sourcing from Byproducts: A Comprehensive Review of Bioactive Molecules in Agri-Food Waste (AFW) Streams for Valorization and Sustainable Applications. Bioresour. Technol..

[19] Carpentieri S., Soltanipour F., Ferrari G., Pataro G., Donsì F. (2021). Emerging Green Techniques for the Extraction of Antioxidants from Agri-Food By-Products as Promising Ingredients for the Food Industry. Antioxidants.

[20] Zhao X., Wang Y., Chen X., Yu X., Li W., Zhang S., Meng X., Zhao Z.-M., Dong T., Anderson A. (2023). Sustainable Bioplastics Derived from Renewable Natural Resources for Food Packaging. Matter.

[21] Jaski A.C., Schmitz F., Horta R.P., Cadorin L., da Silva B.J.G., Andreaus J., Paes M.C.D., Riegel-Vidotti I.C., Zimmermann L.M. (2022). Zein—A Plant-Based Material of Growing Importance: New Perspectives for Innovative Uses. Ind. Crops Prod..

[22] Deng L., Li Y., Zhang A., Zhang H. (2020). Characterization and Physical Properties of Electrospun Gelatin Nanofibrous Films by Incorporation of Nano-Hydroxyapatite. Food Hydrocoll..

[23] Khumkomgool A., Saneluksana T., Harnkarnsujarit N. (2020). Active Meat Packaging from Thermoplastic Cassava Starch Containing Sappan and Cinnamon Herbal Extracts via LLDPE Blown-Film Extrusion. Food Packag. Shelf Life.

[24] Wang P., Li Y., Zhang C., Que F., Weiss J., Zhang H. (2020). Characterization and Antioxidant Activity of Trilayer Gelatin/Dextran-Propyl Gallate/Gelatin Films: Electrospinning versus Solvent Casting. LWT.

[25] LaChance A.M., Hou Z., Farooqui M.M., Carr S.A., Serrano J.M., Odendahl C.E., Hurley M.E., Morrison T.E., Kubachka J.L., Samuels N.T. (2022). Doctor-Blade-Assisted Casting for Forming Thin Composite Coatings of Montmorillonite and Poly(Vinyl Alcohol). Ind. Eng. Chem. Res..

[26] Othman S.H., Kahar N.S., Nordin N., Ahmad Shapi’i R. (2023). Properties and Food Packaging Applications of Solvent Casting-Made Starch-Based Films Incorporated with Essential Oils: A Review. Starch-Stärke.

[27] Martins J.C.L., Garcia J., Guimarães R., Gouvinhas I., Alves M.J., Saavedra M.J. (2026). A Critical Review of Emerging Solutions for Food Packaging: Opportunities and Challenges. Foods.

[28] Ferreira P.S., Ribeiro S.M., Pontes R., Nunes J. (2024). Production Methods and Applications of Bioactive Polylactic Acid: A Review. Environ. Chem. Lett..

[29] Jiang W., Du Y., Huang C., Ji Y., Yu D.-G. (2023). Electrospun Zein Nanofibers: From Food to Food. ES Food Agrofor..

[30] Muthukrishnan L. (2022). An Overview on Electrospinning and Its Advancement toward Hard and Soft Tissue Engineering Applications. Colloid Polym. Sci..

[31] Zhao L., Duan G., Zhang G., Yang H., He S., Jiang S. (2020). Electrospun Functional Materials toward Food Packaging Applications: A Review. Nanomaterials.

[32] Mir S.A., Dar B.N., Wani A.A., Shah M.A. (2018). Effect of Plant Extracts on the Techno-Functional Properties of Biodegradable Packaging Films. Trends Food Sci. Technol..

[33] Pettinato M., Trucillo P., Campardelli R., Perego P., Reverchon E. (2020). Bioactives Extraction from Spent Coffee Grounds and Liposome Encapsulation by a Combination of Green Technologies. Chem. Eng. Process.-Process Intensif..

[34] Li J., Pettinato M., Casazza A.A., Perego P. (2022). A Comprehensive Optimization of Ultrasound-Assisted Extraction for Lycopene Recovery from Tomato Waste and Encapsulation by Spray Drying. Processes.

[35] Re R., Pellegrini N., Proteggente A., Pannala A., Yang M., Rice-Evans C. (1999). Antioxidant Activity Applying an Improved ABTS Radical Cation Decolorization Assay. Free Radic. Biol. Med..

[36] Duba K.S., Casazza A.A., Mohamed H.B., Perego P., Fiori L. (2015). Extraction of Polyphenols from Grape Skins and Defatted Grape Seeds Using Subcritical Water: Experiments and Modeling. Food Bioprod. Process..

[37] Anguelova T., Warthesen J. (2000). Lycopene Stability in Tomato Powders. J. Food Sci..

[38] Drago E., Pettinato M., Campardelli R., Firpo G., Lertora E., Perego P. (2022). Zein and Spent Coffee Grounds Extract as a Green Combination for Sustainable Food Active Packaging Production: An Investigation on the Effects of the Production Processes. Appl. Sci..

[39] (2012). Plastics-Determination of Tensile Properties-Part 1: Test General Principles.

[40] Bisharat L., Berardi A., Perinelli D.R., Bonacucina G., Casettari L., Cespi M., AlKhatib H.S., Palmieri G.F. (2018). Aggregation of Zein in Aqueous Ethanol Dispersions: Effect on Cast Film Properties. Int. J. Biol. Macromol..

[41] Hosseini S.F., Rezaei M., Zandi M., Farahmandghavi F. (2015). Fabrication of Bio-Nanocomposite Films Based on Fish Gelatin Reinforced with Chitosan Nanoparticles. Food Hydrocoll..

[42] Drago E., Campardelli R., Lagazzo A., Firpo G., Perego P. (2023). Improvement of Natural Polymeric Films Properties by Blend Formulation for Sustainable Active Food Packaging. Polymers.

[43] Cerqueira M.A., Souza B.W.S., Teixeira J.A., Vicente A.A. (2012). Effect of Glycerol and Corn Oil on Physicochemical Properties of Polysaccharide Films—A Comparative Study. Food Hydrocoll..

[44] Udana Eranda D.H., Chaijan M., Castro-Muñoz R. (2025). Current Advances in Surface Wettability in Food Packaging Materials: Strategies, Methods and Future Trends. J. Food Eng..

